# Machine Learning Approach for Prediction of Hematic Parameters in Hemodialysis Patients

**DOI:** 10.1109/JTEHM.2019.2938951

**Published:** 2019-10-04

**Authors:** Cristoforo Decaro, Giovanni Battista Montanari, Riccardo Molinari, Alessio Gilberti, Davide Bagnoli, Marco Bianconi, Gaetano Bellanca

**Affiliations:** 1 Department of EngineeringFerrara University 9299 44122FerraraItaly; 2 MIST E-R40129BolognaItaly; 3 Tecnoideal s.r.l.41037MirandolaItaly; 4 Medica s.p.a41036MedollaItaly; 5 CNR-IMM-UOS di Bologna40129BolognaItaly

**Keywords:** Artificial neural network, hematocrit, hemodialisys, machine learning, non-invasive, oxygen saturation, SVM, visible spectroscopy

## Abstract

*Objective:* This paper shows the application of machine learning techniques to predict hematic parameters using blood visible spectra during ex-vivo treatments. *Methods:* A spectroscopic setup was prepared for acquisition of blood absorbance spectrum and tested in an operational environment. This setup is non invasive and can be applied during dialysis sessions. A support vector machine and an artificial neural network, trained with a dataset of spectra, have been implemented for the prediction of hematocrit and oxygen saturation. *Results & Conclusion:* Results of different machine learning algorithms are compared, showing that support vector machine is the best technique for the prediction of hematocrit and oxygen saturation.

## Graphical abstract

This paper shows the application of machine learning techniques to predict hematic parameters using blood visible spectra during ex-vivo treatments. Results of different machine learning algorithms are compared, showing that a support vector machine is the best technique for the prediction of hematocrit and oxygen saturation.

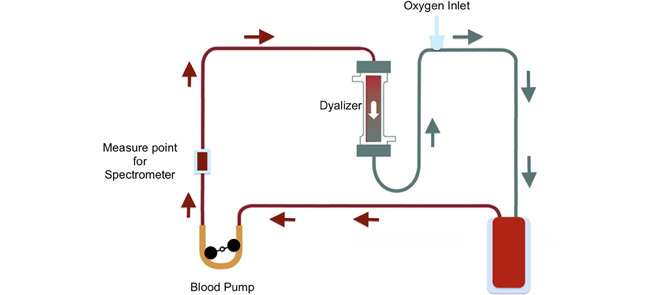

## I. Introduction

The aim of this paper is to present a machine learning approach for estimation of hematic parameters using spectroscopic techniques. There are several applications of opto-electronic sensors for monitoring blood [1], [2], examples of systems for monitoring blood levels during ex-vivo treatments, such as dialysis [3], [4] or modern cardiac surgery [5], are widely reported. A common non invasive technique exploits photo-diode arrays for evaluation of blood parameters, but it provides a limited amount of information, such as oxygen saturation and hematocrit.

Spectroscopy has different applications in biomedical fields, but it is mostly used in diagnostics [6] and therapy [7]. The use of visible spectroscopy for hematic analysis is however a promising approach, because absorbance spectra of blood contain a lot of information such as hematocrit, oxygen saturation, but also platelets [8] and glucose [9] concentrations, allowing the possibility to significantly increase the amount of parameters to be monitored.

Moreover, mini-spectrometers are already available on the market and thanks to their portability, reliability and size it becomes possible to realize a low cost setup for collecting spectra, which forms the database for machine learning implementation. Once machine learning models are trained (e.g. fitted on training data), then they can be used for predictions during dialysis treatment or surgical operations.

Periodic dialysis is commonly performed on patients with end stage kidney failure; this treatment filters wastes and extra water, restoring safe levels of chemicals such as potassium and sodium; it also helps in controlling blood pressure.

Hemodialysis is the most common dialysis treatment; it is an extra corporal (ex-vivo) technique whose main disadvantage is the length of the treatment, which should be also repeated about 4 time a week per patient [10]. The level of hematic parameters, such as hematocrit and oxygen saturation, need to be continuously monitored during the treatment, because patients may suffer hypotension, muscle cramps and lightheadedness.

Moreover, monitoring hematocrit and oxygen saturation is time-saving for patients and significantly improves dialysis efficiency [11].

Hematocrit (Hct) is the ratio between corpuscular part of blood volume and its total volume. It has been already proved that standard values of hematocrit improve the quality of life and reduce risk of mortality in hemodialysis patients [12]. Standard values of hematocrit range from 47 up to 52 in male and from 42 up to 47 in female.

Oxygen saturation (*sO*_2_) of blood is the ratio between the concentration of hemoglobins which have formed a chemical compound with oxygen, called oxy hemoglobin, and the total concentration of hemoglobin. In human blood, standard values of oxygen saturation are above 96%; patients with level of *sO*_2_ lower than 90% are affected by hypoxia, which could be symptom of diseases like asthma or lungs tumor [13].

This study shows the results of two different machine learning techniques, support vector machine and artificial neural networks, for prediction of Hct and *sO*_2_. The dataset, needed for training the two algorithms, is realized by reproducing a real hemodialysis treatment. In this phase, inputs are stored and then preprocessed to create a large dataset.

## II. Machine Learning

Machine learning [14] is a field of artificial intelligence, which exploits statistical techniques, to give a computer system the ability to learn. Fundamental steps of machine learning algorithms are:

1) Learning from a large dataset
2) Generalizing the problem
3) Making predictions on new data

In this paper, two different machine learning techniques, support vector machine (SVM) and artificial neural network (ANN), have been implemented. In literature, there are a lot of examples of support vector machine [15] and artificial neural networks applied for biomedics [16]. On this field, research is mostly focused on automatic diagnosis of different diseases, for example heart activity [17], diabetic retinopathy [18] and sleep apnoea [19]. In the next parts of this section, the two investigated approaches (SVM and ANN) are briefly described and some parameters, commonly used to evaluate their performances, are introduced. SVM and ANN were trained with the same dataset and compared in terms of performance and prediction accuracy. Since both inputs and outputs are provided to the algorithms, the prediction of hematic characteristics is a typical supervised regression task. Dataset must represent different possible combinations of inputs and outputs. It is therefore very important to collect a large dataset with significant measures to train accurate models.

Machine Learning algorithms have many parameters; some are learned during the training phase, while others are initialized before training: these are called hyperparameters. The optimization of hyperparameters has a significant impact on the performance of the model.

### A. Support Vector Machine

Support vector machine (SVM) [20] is one of the most common machine learning technique thanks to its versatility. SVM is a supervised statistical technique which supports both classification and regression problems, with linear or non-linear approach via kernel methods. It finds the best hyperplane which maximizes the inter-distance among the points belonging to the different classes. These hyperplanes represent the decision boundaries allowing categorization of new inputs into one category. Even if SVM algorithm is commonly used for classification, it can also be used for regression. SVM regression tries to fit as many inputs as possible on each hyperplane avoiding misclassification.

The generalization of the hyperplane separation implemented with SVM can be regularized by the C parameter. C is the penalty factor assigned to misclassify data points. When C is small, the algorithm is more tolerant to misclassification, when C is large, the algorithm heavily penalized misclassified data. In this work, Radial Basis Function (RBF) was selected as kernel algorithm to predict hematic parameters, instead of linear kernel which did not provide high accuracy. RBF is the most common non linear SVM kernel and it is characterized by the hyperparameter $\gamma $ [21].

RBF kernel function is equal to:

\begin{equation*} k(x,x^{\prime }) = exp \left ({- \frac {|| x - x^{\prime }||^{2}}{2\sigma ^{2}} }\right)\tag{1}\end{equation*}

which is often rewritten as:

\begin{equation*} k(x,x^{\prime }) = exp (- \gamma || x - x^{\prime }||^{2})\tag{2}\end{equation*}

where $\gamma = \frac {1}{2 \sigma ^{2}}$.

$||x - x^{\prime }||^{2}$ is the squared Euclidean distance between two data points $x$ and $x^{\prime }$.

When $\gamma $ is high, the decision boundary is more affected by individual data points and could lead to overfitting. The optimization of C and $\gamma $ can improve the accuracy of the algorithm, but tuning them is a trade-off. In fact, larger values of C determine smaller-margin hyperplanes; conversely, a very small value of C will cause a larger-margin separating hyperplane. At the same time, when $\gamma $ is very small, the model is too constrained and cannot capture the complexity of data; on the other hand, system will not be able to prevent over-fitting if $\gamma $ is too large [21].

### B. Artificial Neural Networks

Artificial neural network (ANN) [22] is a particular class of machine learning techniques. The name is inspired by biological connections of neuron in human brain. Artificial neural networks are data driven algorithms which learn from a dataset of examples and tries to find out hidden functional relations, even if physics is not explicitly provided.

**FIGURE 1.**
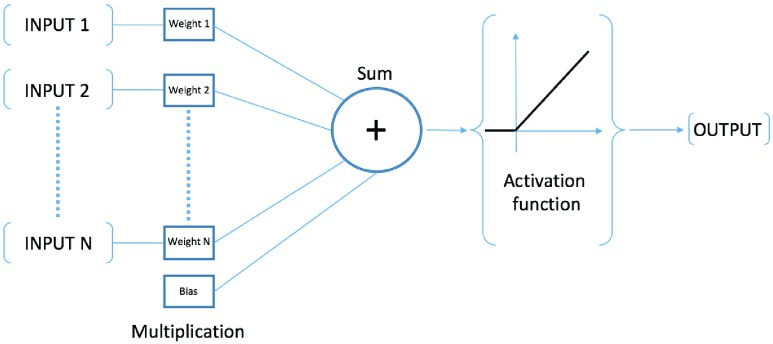
Working principle of an artificial neuron.

They have many different topologies, but all of them are based on their basic block: the neuron. Neurons are processing elements arranged together with different connections. In every neuron, input is associated with a weight and a bias: data passes to structure next level through an activation function. This working principle is schematically represented in figure 1. An artificial neural network is composed by many neurons which are connected together in complex interconnections to solve linear or non-linear problems.

**FIGURE 2.**
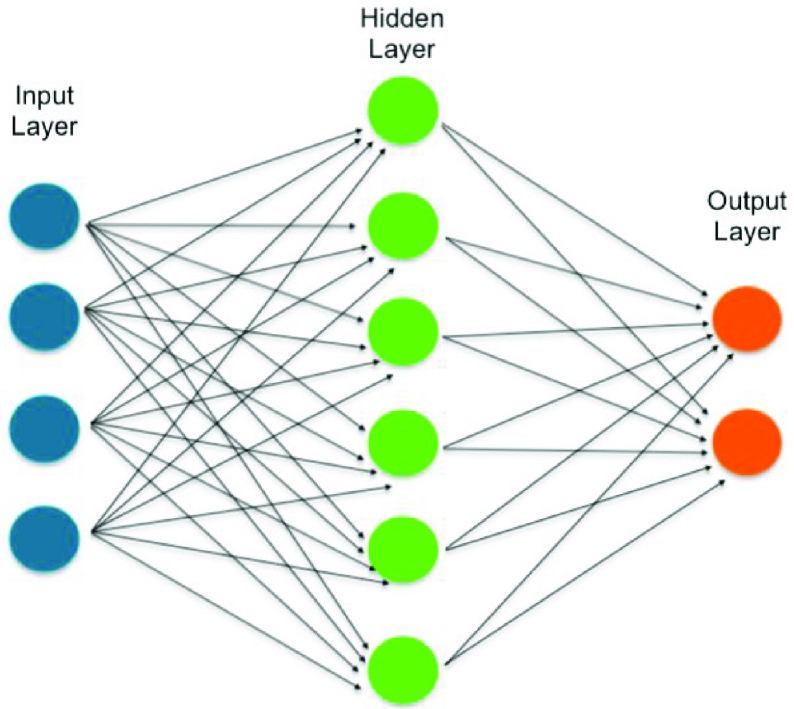
Schematic structure of feedforward propagation.

Feedforward neural network topology was chosen to predict hematic parameters. In a feedforward network, neurons are arranged in layers, with the first layer taking in inputs, one or more middle layers called hidden layers, because they have no connection with external world and the last layer which produces outputs. The information always moves in one direction, there is no loops between neurons and the data never goes backwards. The outputs provided each epochs are compared with the desired values, the error is then fed back through the network during a procedure called backpropagation. Weights are adjusted iteratively to reduce error until some stop criterion is satisfied.

Most of the high computational cost of this method is spent during the training steps. Once artificial neural network is trained for a particular task, then it can be quickly employed to solve similar problems with new data.

In general, there are neither standard steps to determine the best topology of a neural network, nor the best parameters. Basic approach is trial and error starting from the simplest structure and increasing complexity when results are not satisfactory. When the model is reliable and accurate, then it is possible to optimize through modification of some parameters.

The aim is to obtain the most accurate model reducing errors between predicted and target values.

### C. Performance Parameters

In machine learning field, there are different parameters for the evaluation of model’s accuracy.

They all compute the error between desired and predicted values.

The most used performance parameters are:

• Mean Squared Error (MSE) estimated over $n$ samples. It is defined as:
  \begin{equation*} MSE(y,\widehat {y}) = \frac {\sum _{i=1}^{n-1} (y_{j} -\widehat {y_{i}})^{2}}{n} \tag{3}\end{equation*}
• Mean Absolute Error (MAE) estimated over $n$ samples. It is defined as:
  \begin{equation*} MAE(y,\hat {y}) = \frac {\sum _{i=1}^{n-1} |y_{i}-\widehat {y_{i}}|}{n} \tag{4}\end{equation*}
• Coefficient of determination ($r^{2}$). The coefficient of determination is mathematically defined as:
  \begin{equation*} r^{2}(y,\hat {y})=1-\frac {\sum _{i=1}^{n-1} (y_{i} -\widehat {y_{i}})^{2}}{\sum _{i=1}^{n-1} (y_{i} -\tilde {y})^{2}} \tag{5}\end{equation*}
  where $\tilde {y}$ is equal to:
  \begin{equation*} \tilde {y}=\frac {1}{n} \sum _{i=0}^{n-1} y_{i} \tag{6}\end{equation*}
  It provides a measure of how well future samples are likely to be predicted by the model. $r^{2}$ equals to 1 means the model can predict exactly every solution.

In (3) (4) and (5) $y_{1}$, $y_{2}~\cdots ~y_{n}$ are $n$ observed targets and $\widehat {y_{1}}$, $\widehat {y_{2}}~\cdots ~\widehat {y_{n}}$ are the corresponding predicted values.

Calculation of MSE, MAE and $r^{2}$ allows a statistic evaluation of model’s performance, giving a comparison between them.

## III. Methods

### A. Experimental Setup

**FIGURE 3.**
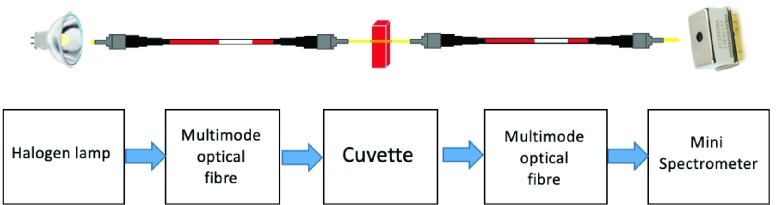
Working principle of experimental setup.

The development of the dataset for the two proposed machine learning algorithms was carried out with spectral acquisition of blood. The hemoglobin absorption and the scattering properties of red blood cells determine a visible spectrum used to be as input for machine learning algorithms, to determine hematic properties. A sketch of the setup for spectra collection is represented in figure 3. It was optimized to record blood transmittance spectra during hemodialysis and it was used, in our study, for developing a large and reliably dataset.

Experimental setup for spectroscopic measurements was composed by:

• a Hamamatsu mini spectrometer, a low cost opto-electronic sensor which collects transmission spectra in the wavelength range from 320 nm to 885 nm and with a resolution of 15 nm [23];
• an halogen lamp used as light source going through the sample;
• multimode fiber optics to connect lamp source and spectrometer;
• a cuvette, which was inserted along the path of blood as the measure point for spectrum acquisition;
• a laptop, which collects and stores data.

**FIGURE 4.**
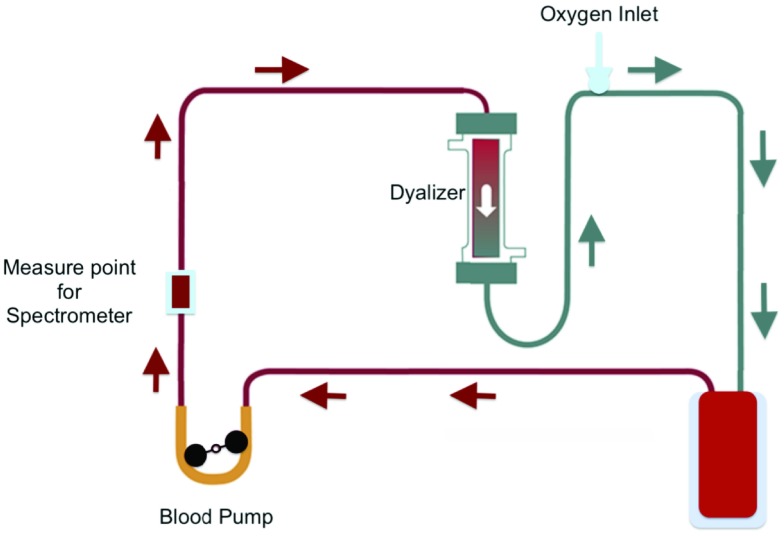
Operational environment for tests.

The described setup was inserted along blood circulation in an operational environment which accurately replicates a real treatment for purification of blood. Setup for experimental tests, as sketched in figure 4, was composed by:

• blood line tubes, where the blood is flowing;
• a blood pump to maintain circulation of blood;
• a dialyzer filter, used to vary hematocrit during blood circulation;
• a cuvette as optical window for spectroscopic measurement;
• an oxygen inlet for introducing oxygen along the path.

### B. Data Collection

In our measurements, 5 simulated dialysis sessions were performed, resulting in a dataset composed by 160 different spectra of animal blood. Every spectrum consists of 287 values, which represent transmittance levels at specific wavelengths. Each spectrum is the average of 100 scans at the highest sensor resolution. All these spectra make the dataset. A reference spectrum of each cuvette was acquired and subtracted from measurements, to remove the signature of the cuvette from the measured spectra.

The input data for machine learning is represented by absorbance. Spectrometer provides the transmittance value of light, Lambert-Beer law was used to evaluate absorbance:

\begin{equation*} T =\frac {I}{I_{0}}\tag{7}\end{equation*}

where:

• $I$ is the light intensity after it passes through the sample
• $I_{0}$ is the reference or initial light intensity

According to Lambert-Beer law, absorption is equal to:

\begin{equation*} A = - log T = - log \frac {I}{I_{0}}\tag{8}\end{equation*}

**FIGURE 5.**
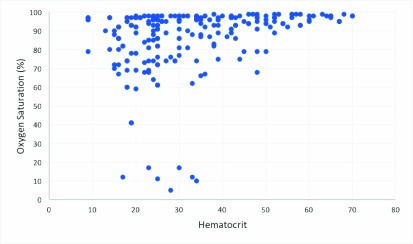
Hct and *sO*_2_ combinations of tested samples.

Standard techniques were used to get the hematocrit and oxygen saturation references; these measures were then collected to build both train and validation sets as targets for machine learning models. Thus, Hct was evaluated through centrifuge to perform blood fractionation, while saturation has been measured through GEM Premiere 3000, a blood gas analyzer of human blood [24]. This is an electrochemical sensor that measures pH, electrolytes and other parameters of blood such as oxygen saturation. GEM Premiere 3000 has a resolution of 1% for *sO*_2_ in the range between 0 and 100% [24]. Different combination, of *sO*_2_ and hematocrit were tested, to provide several possible scenarios. All tested combinations are plotted in figure 5. Samples ranging from 5 up to 100% for the *sO*_2_, and from 9 up to 70 for hematocrit, have been considered. This is a full exhaustive range, because it covers all the possible common situations. However database is not uniform, as most of spectra have *sO*_2_ over 90% and Hct> 20, because this is the most frequent range in hemodialysis patients.

### C. Preprocessing

Support vector machine, artificial neural network algorithms and all other preprocessing operations have been developed in Python 3.7. The targets were considered independently and two different models for SVM and neural network were implemented. The dataset was pre-processed in order to enhance the predictive power of neural networks. Savitzky-Golay’s filter [25] was applied on dataset. This is a digital filter, commonly used in spectroscopy, which removes noise while preserving the characteristics of a signal spectrum [26]. Many machine learning estimators require normalized data to enhance accuracy. Scikit-learn provides different standardization techniques. Robust scaler was chosen for this task. This scaler removes the median and scales the data according to the quantile range, these operations are performed independently on each feature using statistics that are robust to outliers. Normalized dataset was then randomly splitted into two parts: the training set and the test set. The training set, a fraction representing 85% of whole data, was used to fit the models, while the remaining 15% of the data, the test set, was used to evaluate the model’s performances. The split was performed pseudo-randomly because a seed was used to obtain always the same sequence of training and test sets. This is important to compare different models with the same training and test samples.

### D. Training Machine Learning Models

The following Python libraries were used for machine learning:

• Scikit-learn [21] is the package for implementation of support vector machine.
• Keras [27] was used to implement neural network under Tensorflow framework. This is a high level neural networks API, written in Python code.

SVM was fitted with the following hyperparameters:

\begin{align*} C=&10^{3} \\ \gamma=&10^{-3}\end{align*}

k-fold cross validation [21] was used to avoid overfitting in SVM. For each setting of parameter, the k-fold algorithm follows these steps:

• inputs are splitted in k parts (in this case k = 3);
• fitting the algorithm for k-1 parts of inputs (training set);
• evaluation of score for the remaining part (validation set);
• iteration of algorithm for the others k-1 parts;
• evaluation of mean score error for training and validation sets.

k value is an important parameter to fix. Different values of k were tested, but the best result was achieved with k = 3. Artificial neural network is highly penalized by imbalanced dataset. Database is not uniform, because most of spectra have sO2 over 90% and Hct > 20, that is the most frequent range of observation. Oversampling techniques were used to overcame this problem; these technique synthesizes new minority instances between existing minority instances. This approach was applied on training set before fitting process, while the test set remained immutable. The influence of this approach on the final result is under investigation. As previously stated, the neural network was developed by Keras under Tensorflow framework. Artificial neural networks have a set of hyperparameters, as a consequence the optimization process can be long time consuming. Talos [28] library was used in order to fine tune hyperparameters of neural network. Talos is compatible with Keras and it trains neural networks with different hyperparameters finding the best model solution implementing a Grid Search algorithm. In this article, the hyperparameters list has been including: number of hidden layers, learning rate, epochs, activation function and number of neurons. The best solution was finally re-trained by Keras and the final results are here presented. In artificial neural networks, the problem of overfitting was overtaken with early stopping criterion. This method stops the training when the error increases, this is a form of regularization used to prevent overfitting. Keras also provides “reduce learning rate on plateau” technique, it simply adjusts the learning rate while monitoring the loss each epoch.

## IV. Results

As explained in the previous section, the algorithms were trained by supervised learning: targets were provided by centrifuge for Hct and hemo-gas analyzer for *sO*_2_. All the training set of absorbance spectra, measured by spectrometer during dialysis tests, have formed the input data. Hematocrit and oxygen saturation levels of blood samples were predicted by models based on support vector machine and artificial neural networks techniques. Finally, the support vector machine and the artificial neural network were compared through evaluation parameters, to verify the accuracy of both models.

Table 1 shows the overall performance of support vector machine and neural networks for hematocrit prediction on test set.

**TABLE 1:** Performance for Hematocrit

	MSE	MAE	$r^{2}$
SVM	13	2.6	0.95
ANN	14	2.7	0.95

**TABLE 2:** Performance for Oxygen Saturation

	MSE	MAE	$r^{2}$
SVM	0.51	0.55	0.99
ANN	2.4	1.2	0.99

SVM and ANN show similar accuracy performances as reported in table 1.

**FIGURE 6.**
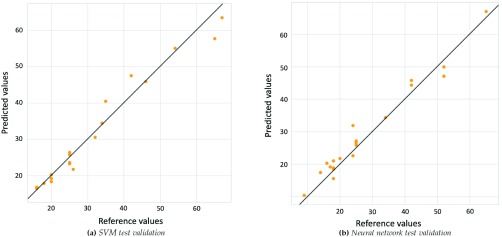
Linear regression model fit on test set for hematocit.

Figure 6 shows the regression plot on the considered test set.

Regression plot analysis function compares actual outputs of two algorithms with the corresponding desired ones (targets).

In this figure, x-axis represents the target values, y-axis represents the predicted values, line represents the perfect fitting between target and predicted values, while scatter points represent test samples. These results show that SVM and ANN are able to implement a model which predicts with good accuracy hematocrit levels using absorbance spectrum inputs. A good prediction accuracy of Hct was achieved by both models ($r^{2} = 95$%). The same analysis was performed for oxygen saturation.

**FIGURE 7.**
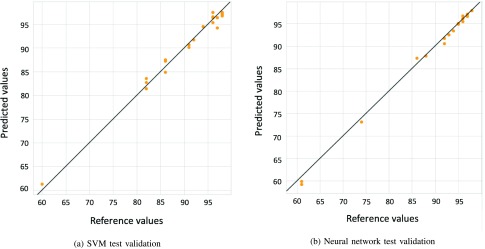
Linear regression model fit on test set for oxygen saturation.

The accuracy of these methods is higher for oxygen saturation than for hematocrit prediction. Results are excellent for both the machine learning-based algorithms, they provide very accurate predictions. For both algorithms, the coefficient of determination is equal to 99%, so models report very high performance. Figure 7 shows accuracy of the models with test data.

Both methods provide efficient predictions, but SVM is, again, the best machine learning algorithm, because MSE and MAE are lower than the ones of ANN.

These results show that both SVM and ANN techniques are able to predict accurately hematocrit and oxygen saturation. The setup here proposed, consisting in the use of mini spectrometer and machine learning techniques, allows results which are comparable with other non-invasive sensors [29], [30] for prediction of hematocrit and oxygen saturation.

## V. Conclusion

This paper shows the application of a machine learning approach combined with a simple and low cost spectroscopic-based setup for monitoring hematic parameters of blood, such as hct and *sO*_2_, during dialysis and other extra corporeal treatments. A support vector machine and an artificial neural network have been implemented and applied to data obtained through spectrometry in the visible and near infrared of different blood samples. Results demonstrate that SVM and ANN models achieved good learning performances and both show the ability to learn relationship between input and targets. In term of accuracy, the most promising algorithm is SVM, but both machine learning methods are able to elaborate accurate predictive models. With respect to other non-invasive techniques which use only few punctual data of the spectrum and which perform the measurements with linear calibration techniques, the advantages of the proposed approach are: high robustness to external light noise due to the optimization of the setup and to the electrical noise thanks to the post processing operations. Machine learning provides general models which does not require calibration. General models mean to have wider range of measurements for hct and *sO*_2_ than other similar sensors. The availability of data belonging to the whole spectrum, will provide more information than other sensors at comparable cost. The combination of spectrometer and machine learning algorithms shows accurate measurements for hct and *sO*_2_, but further studies are conducting indeed to use the same setup along with machine learning in order to measure other different blood analytes. This could represent a decisive improvement than other similar sensor in market at comparable costs.
